# Effects of the COVID-19 pandemic on nurses’ psychological well being in an emergency room

**DOI:** 10.1590/0034-7167-2022-0171

**Published:** 2022-11-28

**Authors:** Sónia Marisa da Rocha Moreira, Rui Manuel Freitas Novais, Maria de Fátima da Silva Vieira Martins

**Affiliations:** I Hospital Centre of Tâmega e Sousa, Emergency Department. Penafiel, Portugal; II Minho University, Nursing School. Braga, Portugal; III Nursing School of Coimbra, Health Science Research Unit: Nursing. Coimbra, Portugal

**Keywords:** Anxiety, COVID-19, Nurses, Pandemics, Emergency Room, Ansiedade, COVID-19, Enfermeiros, Pandemia, Serviço de Urgência, Ansiedad, COVID-19, Enfermeras, Pandemia, Servicio de Urgencia

## Abstract

**Objectives:**

to assess the level of anxiety of nurses in an emergency room in view of the new Coronavirus and describe the relationship between the degrees of anxiety and their sociodemographic variables.

**Methods:**

quantitative descriptive-correlational study with a sample of 60 nurses. A questionnaire was used as a data collection instrument based on the Hamilton Anxiety Assessment Scale.

**Results:**

the nurses’ average anxiety is mild. A statistically significant relationship was found between anxiety and the variables “sex” and “children”, with women having higher levels of anxiety than men, and nurses who do not have children showing mild, moderate, or severe anxiety.

**Conclusions:**

COVID-19 triggers anxiety in nurses, sometimes at pathological levels. Being female and not having children increase the anxiety experienced. Sex can be considered the determining factor for the level of anxiety experienced.

## RESUMO

**Objetivos:**

avaliar o nível de ansiedade dos enfermeiros de um serviço de urgência em face do novo coronavírus e descrever a relação entre os níveis de ansiedade e as variáveis sociodemográficas.

**Métodos:**

estudo quantitativo descritivo-correlacional com uma amostra de 60 enfermeiros. Recorreu-se à aplicação de um questionário como instrumento de coleta de dados baseando-se na Escala de Avaliação de Ansiedade de Hamilton.

**Resultados:**

o escore médio de ansiedade dos enfermeiros é leve. Verificou-se que existe uma relação estatisticamente significante entre a ansiedade e a variável “sexo” e “filhos”, com as mulheres apresentando níveis de ansiedade superiores aos homens e os enfermeiros sem filhos apresentando níveis de ansiedade leve, moderada ou severa.

**Conclusões:**

a COVID-19 desencadeia ansiedade nos enfermeiros, por vezes a níveis patológicos; e ser do sexo feminino e não ter filhos a aumenta. É possível afirmar que o sexo foi o fator determinante para o nível de ansiedade experimentado.

## RESUMEN

**Objetivos:**

evaluar nivel de ansiedad de enfermeros de un servicio de urgencia frente al nuevo coronavirus y describir la relación entre niveles de ansiedad y variables sociodemográficas.

**Métodos:**

estudio cuantitativo descriptivo-correlacional con una muestra de 60 enfermeros. Recorrido a la aplicación de una encuesta como instrumento de recolecta de datos basándose en la Escala de Evaluación de Ansiedad de Hamilton.

**Resultados:**

el escore mediano de ansiedad de los enfermeros es leve. Verificado que hay una relación estadísticamente significante entre la ansiedad y la variable “sexo” e “hijos”, con mujeres presentando niveles de ansiedad superiores a los hombres y los enfermeros sin hijos presentando niveles de ansiedad leve, moderada o severa.

**Conclusiones:**

la COVID-19 desencadena ansiedad en los enfermeros, por veces a niveles patológicos; y ser del sexo femenino y no tener hijos la aumenta. Es posible afirmar que el sexo fue el factor determinante para el nivel de ansiedad experimentado.

## INTRODUCTION

The disease caused by the new coronavirus - Coronavirus Disease 2019 (COVID-19) was first reported in Wuhan, Hubei Province, China, in December 2019. Given its massive spread to hundreds of countries, the World Health Organization declared it a pandemic on 11 March, 2020, constituting one of the largest public health emergencies experienced in human history^(1)^. In Portugal, the first cases of infection were reported on 2 March, 2020^(2)^. Currently, there are more than 240 million cases of COVID-19 and nearly 5 million deaths since the first reported case in China^(3)^.

While thousands of people around the world stayed home to protect themselves and stop the COVID-19 transmission chain, nurses did precisely the opposite. They went to hospitals, namely into emergency rooms, exposing themselves to the risk of contagion. Many were infected and some died as a result of COVID-19^(4)^. Consequently, emergency room nurses live in continuous tension and anxiety, as they experience greatly increased work hours as they attempt to respond to the sudden growth of diseased people^(5)^. There is still little knowledge about COVID-19, however, it is generally agreed that it is highly infectious, and serious cases lead to a severe acute respiratory syndrome that can quickly cause death. These facts trigger anxiety and insecurity in nurses who are directly exposed to the virus by getting in contact and treating infected individuals^(6)^.

Nurses are considered world-leading frontline health professionals^(7)^, and their professional context strongly exposes them to the risk of contamination^(8)^ due to their contact with infected individuals, which triggers insecurity and anxiety^(9)^. Thus, emotional distress drastically interferes with the well-being of nurses^(10-11)^. In this regard, several elements have negative repercussions on the well-being of nurses, generating anxiety for them. Among these, stand out the high risk of being infected, with the consequence of manifesting the disease or even dying; the risk of infecting other individuals, including family members; exposure to death in a much larger scale; the feeling of helplessness in the face of the situation; social distancing from friends and family; and the excess workload^(4,12-13)^.

The feeling of losing the control of the situation, social exclusion, and the stigma for being associated with the disease also contribute to this^(7)^. At the same time, the lack of objective knowledge about the disease and of a specific drug to treat it, the nonexistence of a vaccine during its initial stages, in addition to the constant mutations of the virus, produce feelings of fear and anguish in nurses who are forced to abruptly change their daily lives, which causes them physical and emotional exhaustion, negatively affecting their well-being^(11)^. In view of the lack of information, uncertainty, often contradictory speculation, and of the size of this pandemic, more than 90% of the frontline nurses revealed that they were not fully prepared for the care to be practiced in the context of COVID 19. On the other hand, previous studies carried out in the face of other diseases, such as Influenza A (H1N1) and Ebola, show that 75% of nurses showed preparation and willingness to perform their professional practice^(13)^.

Health professionals show a lack of knowledge about infectious diseases that pose a threat to global health^(14)^. Thus, COVID-19 generates anxiety, with nurses being among the most affected^(11)^. Anxiety and its symptoms cause suffering to the person affected, frequently impairing their social role, professional performance, or other significant areas of their lives, thus interfering with their quality of life. Anxiety translates a complex set of emotions, and fear is the dominant one^(15)^.

Nurses risk their lives both due to their exposure to COVD-19 and to the negative effects on their emotional balance and consequent well-being, taking the risk, these days, of facing a wave of physical and emotional damage which amounts to a parallel pandemic^(16)^. On the one hand, low levels of anxiety are useful to motivate and excite the person; on the other, constant exposure to anxiety has negative consequences for their mental health and professional performance. When not managed, anxiety has long-term repercussions on the performance and personal and professional satisfaction of nurses^(13)^. Anxiety, when untreated, has significant social and personal implications and can lead to social exclusion, increased use of health services, and impair social and family relationships^(12,17-18)^.

Currently, in a reality marked by COVID-19, it is essential to monitor the level of anxiety of nurses to characterize their emotional state and track emotional problems. This monitoring allows the implementation of measures aimed at reducing the level of anxiety, thus avoiding consequences on their physical and emotional well-being^(13)^. Nurses should be warned to be aware of their emotions. When persistent signs of anxiety are overlooked, these can evolve from mild emotional dis-tress to more severe mental disorders^(8)^.

The consequences of this pandemic on the well-being of nurses need to be addressed as an integral part of their response, anticipating situations where emotional disarray may occur, to mitigate short- and long-term damage^(5)^. The first studies on COVID-19 focus on the diagnosis, treatment, transmission, and prevention of the disease. It is important not only to understand the consequences of COVID-19 as a disease, but also its consequences on the short, medium, and long-term emotional well-being of nurses, who, day after day, for almost two consecutive years, cannot be absent from their struggle against this pandemic.

In Portugal, Pinho et al.^(19)^ assessed the stress, anxiety and depression reduction strategies of Portuguese nurses during COVID-19. In the results obtained, 54.3% of the nurses showed anxiety towards COVID-19. Also, the Health Promotion and Prevention of the Non-Transmissible Diseases Department from the *Instituto Nacional de Saúde Doutor Ricardo Jorge*, together with the Institute of Environmental Health of the Faculty of Medicine at *Universidade de Lisboa* and the Portuguese Society of Psychiatry and Mental Health conducted a study entitled “Mental health in times of the COVID-19 pandemic”, whose report, published in October 2020, revealed that about 42% of nurses have moderate to severe anxiety, and nurses caring for COVID-19 infected patients have 2.5 times higher risk of developing emotional damage compared to those not caring for these patients. In our study, whose entire sample was formed by nurses who cared for infected patients, 35% showed moderate to severe anxiety. These data are suggestive of anxiety in regard to COVID-19 as a global phenomenon to all nurses.

In other countries, we found some studies that considered nurses as a vulnerable group to experience psychological distress due to COVID-19^(20)^. COVID-19 generates anxiety in nurses as they are directly exposed to the virus by getting in contact and treating infected individuals. Fear for their own health and the risk of infecting other individuals, including family members, are the most common reasons for anxiety^(4-6,9-10,13,16,20-27)^. Labrague and Santos^(13)^ concluded that over 90% of frontline nurses were not fully prepared to provide care in the context of COVID-19, do not fully grasp the disease, and lack knowledge about it, a shortcoming that is a predictor of anxiety common to all professional classes^(6)^. Difficulties at work such as low productivity, medication errors, needle injuries, and decreased patient satisfaction arise when the nurses’ mental well-being is affected, which jeopardizes the quality of care and the patients’ safety. In this context, the resilience of nurses becomes important and translates into elasticity and recovery capacity, allowing to maintain psychological balance and resist adversity^(13)^.

This study is important because there are insufficient studies in Portugal, and the researchers’ want to contribute to improve the quality of nursing care in the emergency room during the period of the COVID-19 outbreak, considering the well-being of nurses.

## OBJECTIVES

To evaluate the level of anxiety of nurses in the emergency room in relation to COVID-19 and to describe the relationship between the level of anxiety of emergency room nurses and sociodemographic variables (sex, age group, cohabitation, and children).

## METHODS

### Ethical aspects

The study was conducted according to the guidelines of the Declaration of Helsinki, the Oviedo Convention, and the General Data Protection Regulation. It was approved by the Ethics Committee at the *Universidade do Minho* and by the Ethics Committee of the Hospital Centre. Informed consent was obtained from all subjects involved in the study.

### Design, period, and place of study

This is a descriptive and correlational study, with a quantitative approach, developed according to the recommendations of the STROBE checklist. The data collection was carried out in February 2021 in an adult emergency room of a Hospital Centre in northern Portugal.

### Population and sample: inclusion and exclusion criteria

The sample was composed by nurses working in the emergency service. We opted for a non-probabilistic and convenience sampling technique. From a population of 73 nurses, a sample of 60 was formed.

The inclusion criteria were (i) working in an adult medical-surgical emergency room; (ii) caring for people in critical condition with suspected or confirmed COVID-19 diagnoses. The exclusion criteria was working in a paediatrics medical-surgical emergency room.

### Study protocol

Data were collected through a self-administered questionnaire, applied in February 2021, and formed by 2 parts: the first presents questions that enable the socio-demographic characterization of the sample regarding sex, age group, cohabitation, and children; the second was formed by the Hamilton Anxiety Assessment Scale. According to the literature review, this scale is the one that is most accepted by consensus, being the most frequently used in the public domain, and can be self-applied. It consists of 14 items, distributed in two groups. The first group, consisting of 7 items, presents questions related to symptoms of an anxious mood; the second, also with 7 items, addresses the physical symptoms of anxiety. The Likert scale provides five possible answers that vary from 0 to 4, where 0 indicates the absence of a certain symptom, and 4, the maximum intensity of the same. The final score is obtained by the sum of the values assigned by the 14 items of the scale, varying between 0 and 56. This scale classifies anxiety by severity levels, namely normal, mild, moderate, and severe. Higher scores indicate a higher level of anxiety^(28)^. The standards of documentation of the Order of Nurses for mental and psychiatric health in nursing include, in the nursing information systems, as an activity to diagnose the focus “anxiety”, the monitoring of anxiety through the Hamilton Anxiety Assessment Scale. Our reference values in the present study are those applied in this department: values less than or equal to 12 reveal normal anxiety levels, i.e., physiological; values from 13 to 18 indicate mild anxiety; values from 19 to 25, moderate anxiety; and values greater than or equal to 26 show severe anxiety^(29)^.

The printed questionnaires, placed in an individual envelope, were delivered by hand, along with the informed consent, to the service manager, who distributed them to the nurses who accessed the study. After completing it, each participant returned the questionnaire to the manager in the closed envelope provided. Finally, the envelopes were collected by the main researcher. There was no direct contact between the researcher and the participants.

### Analysis of results and statistics

The data collected were analysed with the statistical software Statistical Package for Social Science, (SPSS) version 26. Descriptive statistics made it possible to determine the absolute and relative frequencies, means, and standard deviation. An inferential analysis was also performed to evaluate the relationship between variables. To analyse the distribution of anxiety levels (normal, mild, moderate, and severe) two groups were considered for statistical robustness: anxiety - “no” (normal) / “yes” (mild, moderate, and severe). The limit of statistical significance was established at 0.005; as such, there is a statistically significant difference when p<0.005.

## RESULTS

The health and well-being of nurses are important to a successful emergency service and for the provision of the highest level of clinical care. Globally, the COVID-19 pandemic is having a devastating effect on the mental health and wellbeing of healthcare providers.

60 participants were included in this study. The characteristics of the clinical nurses working in the emergency room are shown in Table 1. 36 (60.0%) were female. Most, 38 (63.3%), are over 30 years of age, with a minimum age of 25 and a maximum of 57 years. Their mean age is 33.5 years, with a standard deviation of 6.6 years around the mean. 54 nurses (90%) live in cohabitation and 25 (41.7%) have children.

**Table 1:** Distribution of sociodemographic characteristics of sample

Variables	n	%	M(SD)	Mn-Mx
Sex			33,5(6,6)	25-57
Female	36	60,0		
Male	24	40,0		
Age group				
<30 years	22	36,0		
≥30 years	38	63,3		
Cohabitation				
No	6	10,0		
Yes	54	90,0		
Children				
No	35	58,0		
Yes	25	41,7		

*n - participants; M - mean; SD - standard deviation; Mn - minimum; Mx - maximum.*

With regard to the level of anxiety among emergency room nurses, 27 (45.0%) revealed normal anxiety levels, 12 (20.0%) mild anxiety, 11 (18.3%) moderate anxiety, and 10 (16.7%) severe anxiety. The mean anxiety score of the 60 nurses was 15.1, with a standard deviation of 9.9 around the mean (Table 2).

**Table 2:** Level of anxiety among emergency room nurses

Level of anxiety ^*^	n	%	M(SD)
<12 (Normal anxiety)	27	45.0	15.1(9.9)
13 a 18 (Mild anxiety)	12	20.0	
19 a 25 (Moderate anxiety)	11	18.3	
≥26 (Severe anxiety)	10	16.7	

* *According to the Mental Health and Psychiatric Nursing Documentation Standards; n: participants; M: mean; SD: standard deviation.*

Regarding the relation between the level of anxiety of emergency room nurses and sociodemographic variables, on which we have based our hypothesis, there is a relationship between the level of anxiety of emergency room nurses and sociodemographic variables, showing that the average anxiety score differed significantly between male and female nurses (p<0.001). Female nurses revealed an average anxiety score higher (19.0) than that of males (9.3). In turn, there are also differences with statistical significance regarding the presence of children (p=0.031), that is, those who do not have children have a mean score indicative of mild anxiety (17.4), while those who have children showed normal anxiety (11.9). There are no significant differences between the mean anxiety score, age group (p=0.108), and cohabitation regimen (p=0.538) (Table 3).

**Table 3:** Relation between the level of anxiety of emergency room nurses and sociodemographic variables

Variables	Anxiety	*p*
	M(SD)	
Sex		<0.001
Female	19.0 (10,2)	
Male	9.3 (5.9)	
Age group		0.108
<30 years	17.8 (10.6)	
≥30 years	13.6 (9.2)	
Cohabitation		0.538
No	17.5 (9.0)	
Yes	14.9 (10.1)	
Children		0.031
No	17.4 (9.0)	
Yes	11.9 (10.3)	

*M - mean; SD - standard deviation; p-value.*

In order to know the distribution of anxiety levels and sociodemographic characteristics, two groups with statistical robustness were considered: “no” (normal anxiety) and “yes” (mild, moderate, and severe anxiety). The data shows statistically significant differences between anxiety and either sex (p<0,001), with 75.0% of women showing mild, moderate, or severe anxiety, compared to men, among whom only 25.0% have these anxiety levels. There is no statistically significant difference between anxiety and either age group (p=0.306) and living arrangements also show no difference (p=0.681). Regarding the variable “children”, there is a statistically significant difference between anxiety and having children or not (p=0.002). 71.4% of those who do not have children have mild, moderate, or severe anxiety, while that is true for only 32.0% of those who do have children (Table 4).

**Table 4:** Distribution of anxiety levels according to sociodemographic characteristics

Variables	Anxiety				*p*
	Normal		Mild, moderate, or severe		
	n	%	n	%	
Sex					<0.001
Female	9	25.0	27	75.0	
Male	18	75.0	6	25.0	
Age group					0.306
<30 years	8	36.4	14	63.6	
≥30 years	19	50.0	19	50.0	
Cohabitation					0.681^*^
No	2	33.3	4	66.7	
Yes	25	46.3	29	53.7	
Children’s					0.002
No	10	28.6	25	71.4	
Yes	17	68.0	8	32.0	

n - Participants; p-value;

* p-value - Fisher’s exact test.

## DISCUSSION

Although the average anxiety score presented by nurses is 15.1 (SD=9.9), nurses are considered to be a vulnerable group in regard to experiencing psychological distress in the face of COVID-19^(20)^. Faced with an unprecedented pandemic, the average anxiety score obtained is categorized as mild, being consistent with another study^(30)^, where the authors reported that nurses, in their daily life, use defense mechanisms to maintain their emotional balance in the face of the adversities of the profession. In addition to the use of these mechanisms, the development of individual resilience can be justified as a strategy to face adverse and stressful situations^(31)^. Resilience contributes greatly for nurses to be able to withstand the weight of anxiety that this pandemic entails^(32-33)^. This opinion is corroborated by other authors^(9,23)^, who point to the strengthening of nurses’ resilience as a pillar to combat their anxiety. The greater the resilience of nurses, the lower the anxiety experienced^(13)^.

Health professionals have a commitment to work and an apparent innate resilience to potentially traumatic events. In this case, nurses have a sense of responsibility and a spirit of mission towards the profession and, in the face of COVID-19, they demonstrate it, while also showing a sense of patriotism and dedication^(9)^. It is also important to praise the commitment, conscientiousness, and compassion they have towards any situation, especially in the face of COVID-19, putting their lives at risk in the performance of their duties^(23,34)^.

In addition to the fact that the mean level of anxiety presented was mild, it was also found that 45.0% of nurses have normal anxiety, that is, physiological anxiety. Since anxiety is intrinsic to all people, in this situation, it functioned as a warning sign for nurses, leading them to develop coping mechanisms that make their anxiety adaptive and temporary^(15)^. However, we cannot devalue normal or mild anxiety levels^(35)^, even when anxiety symptoms do not meet the criteria that numerically indicate a disorder, they can also cause emotional distress and health problems.

In view of COVID-19, emotional suffering expressed through anxiety is ubiquitous^(10)^. Of all nurses in the sample, 55% had altered anxiety levels, among which stand out: 20.0% mild anxiety, 18.3% moderate anxiety and 16.7% severe anxiety. Hu et al.^(9)^ studied the anxiety of frontline nurses and obtained results aligned with those of the present study; specifically, about 45% of nurses suffered from anxiety, with 14% presenting severe levels of anxiety.

In this study, we found that there are statistically significant relations between anxiety and sex (p<0.001). 75% of women have mild, moderate, or severe anxiety, a higher percentage when compared to men, as only 25% of them present these anxiety levels. The mean anxiety level of women is moderate (average score of 19.0), compared to that of men, which is normal (average score of 9.3). This relationship is widely known and corroborates data from the American Psychiatric Association, that informs us that, in regard to sex, anxiety is more frequent in females than in males, at the ratio of 2:1. Women have a higher prevalence of anxiety when compared to men, and generalized anxiety disorder is one of the most frequent conditions^(36)^. Other authors^(17)^ also report that women are almost twice as likely to develop anxiety than men, and add that, in addition to the prevalence of anxiety disorders being about two times higher in women than in men, this difference also manifests itself in the severity of symptoms and treatment efficacy^(37)^.

These results echoed several studies that also found statistically significant differences in relation to sex, regarding the prevalence rate of anxiety, which is higher in women, justifying the sex difference already established in the literature for anxious symptoms^(19,24)^.

Regarding the age group, no statistically significant differences were found (p=0.306). The mean age at the onset of anxiety disorder is 30 years^(15)^, and individuals under 35 years are predictors of the risk of anxiety^(17)^. The average age of this study’s sample is 33.5 years. Regarding age, the study sample is part of the age group where the predisposition to develop anxiety is higher, according to what is described in the literature.

For the variable cohabitation, no statistically significant differences in anxiety levels were found between those who lived alone and those who did not(p=0.681). This result can be justified by the duality found in the literature regarding this aspect. On the one hand, nurses face the fear of infecting other individuals, including family members, and are often advised to distance themselves from their social environment^(4-8)^. In this case, living alone wards off this fear, so it lessens an anxiety factor. On the other hand, it is important to emphasize the importance of social and family support in reducing nurses’ anxiety levels through feelings of understanding, respect, encouragement, empathy, and courage, that lead to more optimistic feelings and, consequently, improves response mechanisms in the face of the adversity caused by COVID-19^(26)^. The support of family members contributes positively to the health and emotional well-being of nurses, helping them to better manage and face the anxiety that comes from professional challenges^(9,13)^. However, COVID-19 is very recent, and there are few references to compare our results. We conclude that these two aspects may justify for the absence of statistically significant associations with this variable.

Regarding children, we found that there is a statistically significant relationship between anxiety and having or not children (p=0.002), with 71.4% of nurses who do not have children presenting mild, moderate, or severe anxiety compared to those who do have children, only 32.0% of whom show these same levels of anxiety. The satisfaction of being a parent may justify a lower prevalence of anxiety among other mental disorders. Persons with children are less likely to develop anxiety because they feel support in the family, which protects them from its development^(38)^. Those who have children organize and use their time better, and taking care of their children is, in addition to a source of gratification, a way to stay farther from the source of anxiety^(39)^. This is evident in our study, since 68% of nurses who have children show normal anxiety levels. The strategy to alleviate the anxiety triggered by COVID-19 is to try to keep the routine as close to the usual as possible. Those who have children have to maintain several daily domestic routines, thus providing less emotional time for COVID-19.

### Study limitations

Collecting data at a single moment may be limiting, and the comparison of the results in more than a moment could have broadened the conclusions on the relationship between the variables. The data were collected in February 2021, and the moment of the research may limit the sample size and the generalization of the results to other periods. The peak experienced by the emergency service studied was in October, November, and December 2020.

### Contributions to the area of Nursing, Health, and Public Policy

There are still few studies on this theme, making it difficult to contextualize and discuss. More time needs to pass before the true impact of this pandemic on nurses’ emotional balance can be determined. Based on scientific evidence, which tells us that the impact of this pandemic on the well-being of nurses may surface later, we suggest that studies on anxiety in this professional class should be conducted in the post-pandemic period, to obtain more robust conclusions about its real impact. We believe that this study may be helpful in this regard.

## CONCLUSIONS

All healthcare professionals have the right to feel safe and protected in their work environment. COVID-19 dealt a severe blow to this premise, revealing itself to be harmful to the physical and emotional well-being of nurses.

This study demonstrates that COVID-19 triggers anxiety in nurses, sometimes at pathological levels, and that being female and not having children increase the anxiety experienced. It was concluded that there is a relation between the level of anxiety and the female sex, which makes it possible to state that the nurses’ sex was the determining factor for the level of anxiety experienced. Being a woman is predictive of higher levels of anxiety, most likely due to the use of less effective adaptive strategies and to having less social support than men, including having more domestic tasks and responsibilities that are cumulative with professional demands. Women have an inherently higher vulnerability to anxiety, and physiologic, neurologic, genetic, and hormonal differences contribute to this. On the other hand, individuals with children are less likely to develop anxiety because they feel support from their family, which protects them from its development. Caring for children implies an adequate organization of time, also being a source of gratification and a way to stay farther from the source of anxiety.

This study also stresses the need to consider and recognize the fears of nurses, thus creating a sphere of stability in the midst of the crisis, and reiterating the need to prevent two pandemics: the current, in the struggle to stop the spread of COVID-19, and the one that will come tomorrow, in the form of the damage to the emotional well-being of nurses caused by the same struggle. We must not fail to take care of those who are providing care today.

It is imperative that the focus of hospital institutions is no longer only caring for the person infected with COVID-19, but also for those who provide this care, as a way to avoid the worsening of the pandemic scenario. We conclude that, even as they show clear signs of physical and emotional fatigue, nurses continue to express the desire to work on the frontlines in the struggle against the pandemic. Identifying anxiety levels in this professional class is paramount to operationalize training programs and to plan and implement intervention plans focused on the well-being of nurses, professionals who are fundamental in combating this pandemic.
